# Study of the Effect of Mulching Materials on Weed Control in Saffron Cultivation in Eastern Morocco

**DOI:** 10.1155/2021/9727004

**Published:** 2021-12-21

**Authors:** Ibtissam Mzabri, Maria Rimani, Khadija Charif, Nourddine Kouddane, Abdelbasset Berrichi

**Affiliations:** ^1^Laboratory for Improving Agricultural Production, Biotechnology and the Environment, Department of Biology, Faculty of Sciences, University of Mohammed First, BP717, Oujda 60000, Morocco; ^2^Laboratory Bioresources, Biotechnology, Ethnopharmacology and Health, Department of Biology, Faculty of Sciences, University of Mohammed First, BP717, Oujda 60000, Morocco

## Abstract

Saffron (*Crocus sativus* L.) is cultivated in many countries for its culinary and medicinal values. The production of saffron is limited by several factors, including weed infestation, which causes damage to the crop in terms of quantity and quality. However, little information is available on the different weed management strategies for saffron cultivation, as most of the strategies implemented are developed for large-scale and conventional agriculture. As a result, they are not applicable or affordable for organic or smallholder farmers, as is the case for saffron cultivation. The objective of this study is to compare the effectiveness of plastic mulching versus mulching in controlling weeds in saffron cultivation in the eastern region of Morocco. During the trial, which was conducted in 2018, the parameters measured correspond, on the one hand, to morphometric measurements and determination of saffron stigma yield and, on the other hand, to the determination of density, dry biomass, and weed control capacity. Compared to the control, mulching reduced the population and dry biomass of the most formidable weeds such as *Cynodon dactylon*, *Aster squamatus*, *Cyperus rotundus*, and *Convolvulus arvensis*. The average stigmata yield from plastic mulch treatment was 9% higher than of the control, and the number of leaves, leaf area, number, weight, and percentage of daughter corms with large diameter were higher for plants grown under mulch. Overall, the results of this study showed that the use of PE (polyethylene) mulch effectively reduced weed populations and improved saffron yield and vegetative growth.

## 1. Introduction

The fluctuation of biotic and abiotic stresses is a key issue for good quality agricultural production [1]. Wheeler et al. [2] reported that this production would be influenced by the preponderant increase in global temperature expected towards the end of the 21^st^ century. The effect of this change, in addition to crop-weed infestation, leads to a complex crop-weed interaction [3]. This interference is considered one of the most important factors in decreasing global agricultural production, especially in organic agriculture, where the use of synthetic chemicals is prohibited. The competition between weeds and crop plants occurs mainly in relation to water, light, nutrients, and space. Similarly, some weeds have an allelopathic effect by releasing toxic substances that can inhibit the germination and growth of many crops [4].

Saffron has been cultivated as a spice since ancient times (−3500 years). It has crossed several continents and civilizations. Throughout history, it has been among the most expensive substances in the world [5]. Although Morocco is a small producer of saffron, its quality is high nationally and internationally.

Saffron production is limited to several factors, including weed infestation because saffron is a small, slow-growing plant that does not provide a well-developed aerial part [6], and the dense and rapid growth of weeds intensifies the damage to the saffron crop.

As a perennial crop, saffron is infested by different types of annual, biennial, and perennial weeds. Rimani et al. [7] classified *Convolvulus arvensis*, *Bromus rubens*, *Lolium perenne*, *Hordeum murinum*, *Isatis tinctoria*, *Malva parviflora*, and *Lamium amplexicaule* among the twelve (12) most problematic weeds of saffron in Morocco.

Weed control is an important aspect to consider when planning the production of any agricultural speculation. Whether done manually, mechanically, or chemically, it represents a considerable burden for the farmer [8].

The chemical method of applying herbicides is one of the most effective weed control methods. Therefore, the use of the chemical method remains expensive and not environmentally friendly [9], especially in Morocco, which adopts organic saffron production, so the use of any chemical input is deemed prohibited.

Manual weeding is the most common method used by Moroccan farmers; however, this practice is time-consuming and labor-intensive, which reduces the profit margin of small- and medium-scale farmers. Similarly, Zhang et al. [10] reported that hand weeding in high-density crops can damage the subsurface and therefore is not always recommended for weed control.

Mulching is one of the most important means of weed control [11, 12]. The main objective of mulching is to prevent light from reaching the weeds and thus stop their growth [13, 14]. In addition, natural mulching can increase soil organic matter [15], improve water use efficiency [16], prevent erosion [17], and change the physical environment of the soil [18]. However, the effectiveness of mulch varies according to the type used, its thickness, environmental factors, the species cultivated, and its production technology [19–21]. Much research has been carried out on the application of organic mulch [20, 22, 23], where the application of citronella mulch resulted in higher yields of herbs and essential oils [24]. In the same way, the efficacy of inorganic mulching has been widely studied on many aromatic and medicinal species such as rosemary, thyme, and lavender [25]. Moreover, Parmar et al. [13] showed superior yield and quality of watermelon produced on PE black film. In saffron cultivation, the application of sawdust mulch significantly improved spice yield and the rate of corm multiplication [26].

It should be noted that little work has been done to verify the effectiveness of these biological weed control methods in the cultivation of saffron in Morocco. One study compared the effectiveness of plastic and organic mulch on the loss of viability of weed seeds associated with saffron cultivation.

## 2. Materials and Methods

### 2.1. Site Characteristics

**Figure 1:**
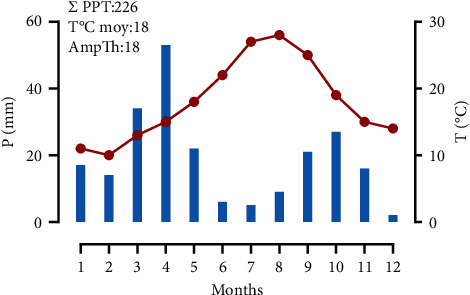
Average monthly meteorological data from the experimental station of the faculty of science in Oujda during the trial period.

The experiment was conducted in the open field at the experimental research station of the Faculty of Science of Oujda, located in eastern Morocco at an altitude of 661 m at 34°39′06 -71″ north and 01° 53′58 -80″ west (GPS BackTrack Bushnell). The climate of the area is semiarid, characterized by temperate winters. During the test period, rainfall was low at 226 mm. The average annual temperature is 18°C, reaching 29°C in summer and 10°C in winter. Saffron water requirements were supplemented by localized drip irrigation during dry periods (Figure 1).

### 2.2. Plant Material

The saffron corms, more than 2.5 cm in diameter, used in this test come from a saffron boat at the experimental research station of the Faculty of Science in Oujda.

### 2.3. Applied Treatments

**Figure 2:**
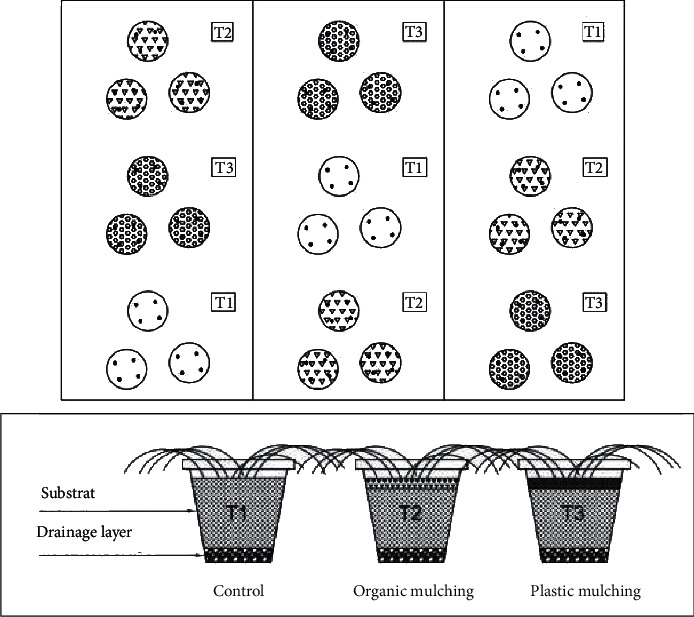
Experimental design in a complete randomized block and the proportions of substrates used in each treatment.

Treatments included organic and plastic mulching. The types of mulch were selected based on the mulches most commonly used by small and medium farmers in agricultural plantation systems. After planting the saffron corms, the leaves from mowing the lawn were applied to a thickness of 3 cm. The required quantity of grass leaves (*Festuca arundinacea*, *Lolium perenne*, and *Agrostis stolonifera*) comes from the operation of mowing the green areas of the Faculty of Science in Oujda. The plastic mulch used is made of black polyethylene with a thickness of 25 microns. The plastic mulch was cut to the size of the pots and fixed to the edges of the pots using soil. Afterwards, holes of 5 cm *∗* 5 cm were applied in the plastic mulch to facilitate the emergence of the saffron shoots. When the pots were filled, the substrate composed of a mixture of peat and sand (2/3 and 1/3 v/v respectively) was prepared, and then a quantity of sheep manure of 180 g/pot was uniformly spread and incorporated into the substrate (10 cm) as a basic amendment to meet the requirements of the crop. The experimental design was a complete randomized block design with three treatments and nine replicates, totaling 27 pots, each closing four saffron plants for a total of 108 plants. There were three treatments: T1: control treatment without mulching, T2: organic mulching (grass cutting), and T3: plastic mulching (Figure 2).

### 2.4. Measured Parameters

#### 2.4.1. Agrofloristic Parameters

The weeds in each pot were identified and classified into monocotyledons and dicotyledons. Weed densities were measured monthly across the entire surface of each pot from October 30, 2018, to April 30, 2019. The total number of weeds is cumulative. The weeds were cut flush with the ground and then kiln-dried at 70°C to constant weight. The dried plants were weighed to measure the dry above-ground biomass. The weed control capacity of the different treatments used was calculated according to the following formula:

(1) $$\begin{array}{c} \text{WSA}\left(\%\right)=\left[\frac{\left(\text{WB Control}-\text{WB Treatment}\right)}{\text{WB Control}}\right]\times 100. \end{array}$$

WSA is the weed control capacity, WB Control is the weed biomass of control treatment, and WB Treatment is the weed biomass of treatment [27].

#### 2.4.2. Durability of Mulching

The durability of the mulch was determined by the percentage coverage of weeds growing through the mulch. Weed damage was rated visually on a five-point scale: no damage, slightly damaged, damaged, severely damaged, and completely damaged.

#### 2.4.3. Agromorphological Parameters of Saffron

Biometric observations (height and number of leaves) were recorded five months after planting. Stigma yield was measured at the end of the saffron harvest after drying the fresh stigmas in the shade for a few days. The parameters of the underground part (number, weight, and diameter of corms) were calculated at the end of the cultivation cycle after digging out the plants.

### 2.5. Data Analysis

The data that met the assumptions of the analysis of variance (ANOVA) were subjected to an ANOVA analysis, using the software “GraphPad Prism for Windows version 7” and the comparison of the means is made by Duncan's test at the 5% significance level. Weed densities were analyzed using a Wilcoxon nonparametric multiple comparison method because these data did not meet the assumptions of normality for ANOVA. The size of the difference between the two groups was tested by Student's *t*-test at the 5% significance level.

## 3. Results

### 3.1. Weed Identification

The results of the floristic survey carried out showed that a total of 20 common weed species were inventoried at the study site. The species classification recorded 6 monocotyledons and 14 dicotyledons. Thus, the 20 species belong to 12 botanical families: Cyperaceae, Asteraceae, Convolvulaceae, Oxalidaceae, Malvaceae, Poaceae, Brassicaceae, Chenopodiaceae, Papaveraceae, Primulaceae, Lamiaceae, and Fabaceae. The densely populated families were Cyperaceae and Poaceae, while the Asteraceae family was the most diverse family. Furthermore, the most dominant weed species were *Cyperus rotundus*, *Conyza* spp., *Convolvulus arvensis*, *Anagallis arvensis*, *Malva parviflora, Cynodon dactylon*, *Chenopodium album*, *Bromus sterilis*, *Aster squamatus*, *Fumaria parviflora*, and *Medicago truncatula* (Table 1).

### 3.2. Effect of Mulch Type on Weed Parameters

**Figure 3:**
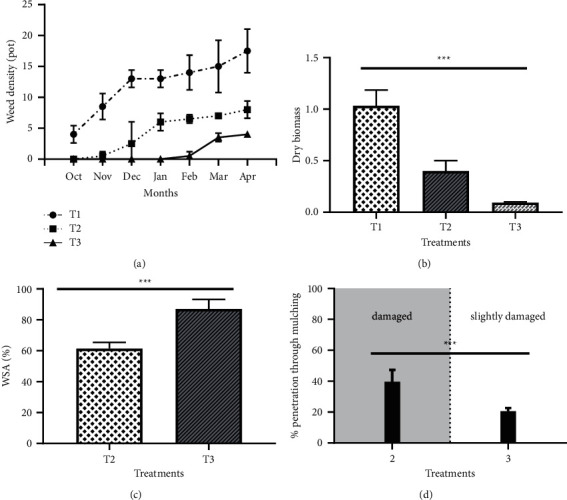
Effects of mulch type on weed parameters. (a) Weed (density/pot), (b) dry weed biomass (g), (c) weed suppression capacity (%), and (d) percentage of weed emergence through the mulch. Data are the average of five measurements. The symbols ^*∗*^,^*∗∗*^, and ^*∗∗∗*^ indicate significant differences at *p* ≤ 0.05, *p* ≤ 0.01, and *p* ≤ 0.001, respectively, at a unidirectional ANOVA and Student's *t*-test.

It is clear that both types of mulching resulted in a significant reduction in weed density (Figure 3(a)). The control (T1) recorded the highest cumulative density, while the minimum cumulative weed density was observed in the case of plastic mulching, which recorded no weeds during the active period of saffron cultivation (October–February). Organic mulching also significantly reduced weed density (*p*=0.004). During the last weed measurement in April, the cumulative number of weeds was lower in the T2 and T3 treatments compared to the control with reductions of 56% and 79%, respectively. Among the species identified, *Convolvulus arvensis* and *Bromus sterilis* had the highest number of individuals in the unmulched treatment. However, *Cyperus rotundus*, independently of mulching, was observed in all treatments (T1, T2, T3). Similarly, dry weed biomass followed the same trend of density with the control (T1) showing the highest biomass (26.3 g/m^2^) followed by organic mulching (10.5 g/m^2^), while plastic mulching came last with the lowest biomass (2.63 g/m^2^), a decrease of 90% compared to the control. These results are confirmed by the ANOVA analysis which showed a very highly significant difference between the three treatments (*p* ≤ 0.001) (Figure 3(b)). Weed control efficacy was dependent on the dry biomass of the weeds, and the maximum control capacity (87%) was recorded for the treatment (T3) compared to a control capacity of 61% for the organic mulch (T2). This difference was very highly significant according to the Student's *t-*test (*p*=0.001) (Figure 3(c)). In terms of durability, plastic mulch was the least damaged and therefore the most durable (Figure 3(d)), although the weeds that had grown through this type of mulch (mainly *Cyperus rotundus*) barely reached 20% of the mulched area while a penetration percentage of 39% was observed in the case of turf leaves which damaged this type of mulch.

### 3.3. Effect of the Type of Mulching on the Agromorphological Parameters of Saffron

**Figure 4:**
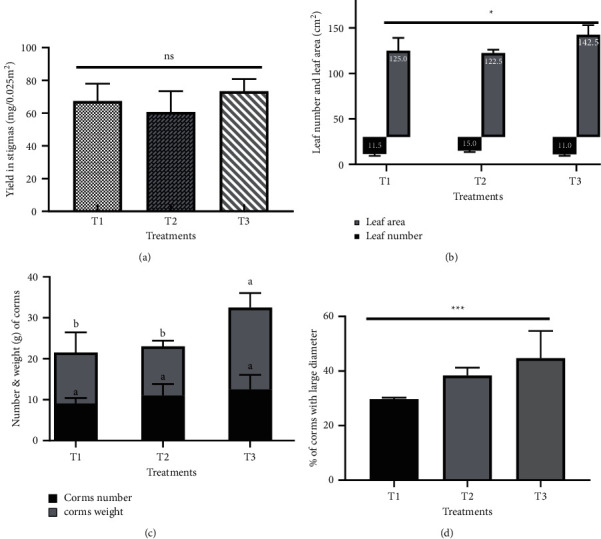
Effects of the type of mulching on the agromorphological parameters of saffron. (a) Saffron stigmata yield, (b) number of leaves and leaf area (cm^2^), (c) number and weight g of daughter corms, and (d) large wire corms (%). The data are the average of five measurements. The symbols ^*∗*^,^*∗∗*^, and ^*∗∗∗*^ indicate significant differences at *p* ≤ 0.05, *p* ≤ 0.01, and *p* ≤ 0.001, respectively, at a unidirectional ANOVA. Different letters indicate significant differences between treatments (*p* < 0.05).

The results showed that mulching significantly influenced certain agromorphological parameters of saffron. The stigmata yield was higher in the case of plastic mulching (1.9 g/m^2^), an increase of 9% compared to the control. However, according to the analysis of variance, this difference is not statistically significant (*p*=0.09) (Figure 4(a)). The maximum number of leaves (15 leaves) was recorded by the application of organic mulch (T2), while the effect of plastic mulch (T3) was similar to that of the control (T1) (Figure 4(b)). Plastic mulch recorded a significantly higher leaf area (142.5 cm^2^) compared to the other treatments (Figure 4(b)). Likewise, the parameters of the underground part were influenced by the type of mulching. In contrast to the number of corms, which did not differ significantly between treatments (*p*=0.12), mulching allowed a significant increase in the weight and diameter of the saffron corms (Figures 4(c) and 4(d)). The largest increase in weight (32.5 g) and diameter of corms (44%) was observed in the case of plastic mulching (T3), with increases of 34% and 35%, respectively, compared to the control treatment. The effect of organic mulching (T2) was also significantly better than the control, which showed a 23% improvement in the percentage of large diameter daughter corms.

**Figure 5:**
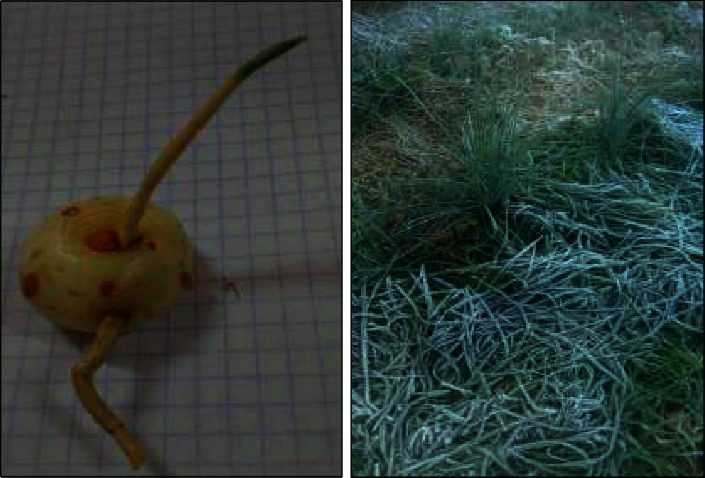
(a) *Cynodon dactylon* damage on saffron corms. (b) Spring frost on mulch.

It should be noted that field observations have shown that organic mulch (turf leaves) spread on the ground increases the risk of frost by acting as an insulator. Organic mulch slows down the absorption of heat by the soil during the day and also slows down its dissipation during the night, thus delaying the warming of the soil. If it is under the flowers during a frost, the risk of damage may increase (Figure 5).

## 4. Discussion

In the present study, the weed flora reported was common in all treatments applied. The results show a dominance of the dicotyledonous class over the monocotyledonous class. Thus, of the nine families encountered, Asteraceae and Poaceae were the main families present in the trial. These results are comparable to those found by various studies on a national scale on either saffron cultivation [7] or other agricultural speculations [28, 29], where the Asteraceae family almost always occupies the first rank in the spontaneous flora of Morocco. This dominance can be justified by the ability of these families to produce high quantities of seeds, by their Mediterranean range and their ability to adapt to unstable and diverse biotopes [7]. This makes the search for management methods, such as solarisation and mulching, to control these species a necessity. Among the problematic species identified, *C. rotundus* was present in all treatments, although, in fairly small numbers, it was the only species that was able to grow through plastic mulch (T3), followed by the tooth dog (*C. dactylon*). These species are considered among the 20 weed species that are difficult to control and cause significant damage to world agriculture [30]. The results found showed that mulching reduced the infestation by this problematic species. A similar result has been proven by other studies [15, 31].

All treatments applied were effective in reducing weed density and dry biomass compared to the control (Figures 3(a), 3(b)). These results are also consistent with those of [32] on sweet potato and [33] on turmeric, which also found the highest number of weeds in the control plots, due to the absence of any physical obstacles and constraints. Thus, the weed seeds found the right conditions for germination and growth. However, the application of organic (T2) or plastic (T3) mulch considerably reduced the weed density with reductions of 56% and 79%, respectively. Similarly, the adoption of different mulching treatments significantly reduced the dry biomass of the weeds, in particular black plastic mulch (T3), which was able to reduce this biomass by 91%, followed by organic mulch (T2) by 61% compared to the control (T1). Our data corroborate previous studies on *Capsicum* [34], *Curcumin* [33], and citrus [35] because of the physical effect of mulching, which inhibits weed germination. Even if this occurs, the conditions under the mulch discourage weed growth, which is deprived of light and initiates photosynthesis [15], especially the black plastic mulch, which has resulted in weed control close to 100%, which suggests that manipulating the row spacing of the saffron crop could inhibit weed germination and growth by reducing incoming energy or changing the spectral composition of the rays [36, 37]. Similarly, studies on saffron have shown that the use of shade cloth contributed to weed control (unpublished results). In addition, results showed that mulching resulted in better control capacity compared to the control. The same finding was reported on tomato and ginger [38]. In this study, the longest lasting effect of weed control was provided by black polyethylene mulch with an average weed coverage of 20% at the end of the saffron crop cycle, whereas the organic mulch based on the leaves of the turf was more damaged following a weed penetration percentage of 39%. In addition, it is important to point out the formation of frost under the turf leaves during the cool period of March and April.

In addition, the results showed that mulching improved the agromorphological parameters of saffron and the dry stigmas yield was higher than the control (Figure 4(a)), which could be explained by optimized soil temperature and moisture, controlled evaporation losses, and availability of nutrients to the plants. These favourable conditions contributed to the production of more, longer, and wider leaves (Figure 4(c)). Similar effects were found by Ibarra-Jiménez et al. [39] on cucumber, Lira Junior et al. [40] on curcumin, and Kaushal et al. [41] on ginger. Moreover, Nyamangara et al. [42] reported that the effect of mulching on yield was greater during dry seasons, indicating the importance of mulching in moisture conservation, especially in areas with lower-than-average annual rainfall, as is the case in the eastern region of Morocco. In addition, the increase in yield in the case of plastic mulching (T3) is partly attributed to an increase in soil temperature and photosynthesis [43]. Optimization of the vegetative parameters (number of leaves, leaf area) following mulch application helped saffron plants translocate and accumulate excess photosynthesis in the underground part, which induced the development of a greater number and weight of corm threads. This effect is all the more important in the case of plastic mulching, which corroborates the work on ginger [44]. In addition, the reduction in weed density reduced weed competition and allowed better aeration for the development of large-diameter of daughter corms. Similar results were reported by Anikwe et al. [45] on groundnut and Thankamani et al. [44] on ginger.

## 5. Conclusion

The results of the present study highlighted the importance of the use of mulching on weed control and the agromorphological parameters of saffron. Better weed control was recorded in the case of the use of plastic mulch, whose maximum suppression was recorded following the use of black plastic mulch (87%). Yield and growth parameters were considerably improved following the use of mulch, whose average yield of stigmas from plastic mulch treatment was 9% higher than that of the control. Likewise, the weight and percentage of daughter corms with a large diameter were higher for plants grown under mulch. In general, the results suggest that black plastic mulch or mulch can provide a viable and effective control compared to the control and therefore could be an alternative for organic farmers to counter weeds in saffron production, especially in the semiarid region of Morocco.

## Figures and Tables

**Table 1:** Floristic survey of weeds and their distribution in the experimental area.

T1		T2		T3	
Weeds	Number	Weeds	Number	Weeds	Number
*Cyperus rotundus*	3	*Cyperus rotundus*	2	*Cyperus rotundus*	1
*Conyza* spp.	*3*	*Conyza* spp.	*2*	*Conyza* spp.	1
*Convolvulus arvensis*	*3*	*Convolvulus arvensis*	*2*	*Convolvulus arvensis*	1
*Cynodon dactylon*	3	*Cynodon dactylon*	2	*Cynodon dactylon*	2
*Bromus sterilis*	3	*Bromus sterilis*	1	*Bromus sterilis*	0
*Anagallis arvensis*	*1*	*Anagallis arvensis*	*2*	*Anagallis arvensis*	0
*Malva parviflora*	1	*Malva parviflora*	1	*Malva parviflora*	0
*Chenopodium album*	1	*Chenopodium album*	0	*Chenopodium album*	0
*Medicago truncatula*	1	*Medicago truncatula*	0	*Medicago truncatula*	0
*Aster squamatus*	1	*Aster squamatus*	1	*Aster squamatus*	1
*Fumaria parviflora*	1	*Fumaria parviflora*	0	*Fumaria parviflora*	0
*Hordeum murinum*	1	*Hordeum murinum*	1	*Hordeum murinum*	0
*Sonchus oleraceus*	1	*Sonchus oleraceus*	0	*Sonchus oleraceus*	0
*Sinapis arvensis*	1	*Sinapis arvensis*	0	*Sinapis arvensis*	0
*Lamium* sp.	1	*Lamium* sp.	0	*Lamium* sp.	0
*Melilotus* sp.	1	*Melilotus* sp.	1	*Melilotus* sp.	0
*Oxalis pes caprae*	1	*Oxalis pes caprae*	0	*Oxalis pes caprae*	0
*Sisymbrium officinale*	1	*Sisymbrium officinale*	0	*Sisymbrium officinale*	0
*Avena sterilis*	1	*Avena sterilis*	0	*Avena sterilis*	0
*Festuca arundinacea*	1	*Festuca arundinacea*	0	*Festuca arundinacea*	0

## Data Availability

The data used to support the findings of this study are available from the corresponding author upon request.
